# Hospital and Institutional News

**Published:** 1915-07-10

**Authors:** 


### HOSPITAL AND INSTITUTIONAL NEWS.

### THE KING'S INVESTITURE: NOTICE.

Medical men who have been included in the recent lists of decorations may be reminded that the Lord Chamberlain is authorised to announce that the King will hold an Investiture at Buckingham Palace on Monday, the 12th inst., at 11.30 A.M. The official statement requests that any gentlemen promoted in or appointed to the Orders of the Bath, Star of India, and Indian Empire, who are awaiting investment, will communicate at once with the Registrar and Secretary, Central Chancery of the Orders of Knighthood, St. James's Palace. Knights Bachelors designate should communicate direct with the Private Secretary, Home Office. All officers who have been awarded decorations during the present war and who have not yet received their decorations are requested to report at once, in writing, to the Secretary, War Office, stating their first Christian name and surname, rank, regiment, etc., address, and the nature of the decoration awarded.

### THE SPIRIT TAX AND THE BRITISH MEDICAL ASSOCIATION.

At the time of writing the report stage of the Finance Bill had not been reached, but it is the present intention of Mr. Bridgeman to renew his efforts to have inserted in the Bill the clause providing for the use of duty-free spirit in hospitals, which met with such surprisingly unfavourable reception at the Committee stage. It is understood that there have been conversations between the sponsors of the proposed clause and those who opposed it, and hope has not been abandoned that a compromise will be arrived at for securing, at any rate, some portion of the benefits which the original clause would have conferred on hospitals. The attitude of the British Medical Association is very difficult to understand, and it is doubtful whether the policy of the Association in this matter is thoroughly representative of the views of its constituents.

### THE RANELAGH RED CROSS FETE.

Our press-day this week also happens to be the occasion of the open-air fête in aid of Red Cross funds, the object of which was described in our columns recently. As we then pointed out, this fête provides the first example of the devotion of the famous Ranelagh grounds, the summer haunt of fashion in London, to the purposes of charity. It is unnecessary to re-emphasise the appositeness of this appropriation. Indeed, by its gift of the grounds the Ranelagh Club receives its national apotheosis, since it is safe to say that on no other occasion during the present summer could there have been the same crowd that was confidently expected to throng them on Wednesday afternoon. The attractions of Ranelagh could not be sufficient to counteract the absence of innumerable members whom work for the State in various departments has kept rigidly away. Again, the fact cannot be escaped that grief has come to many homes, none the less because its occasion has been a glorious sacrifice; and under the shadow of personal loss the thought even of an afternoon at Ranelagh has been hardly welcome. On the occasion of this fête however, the attractions, which by themselves would hardly have been even suggested, have a new and happy regard, for have not all who enjoy them at least the thought that their attendance is not mere pleasure-seeking, but that the natural instinct in that direction has been given a practical working value, since a large attendance means a great accession to the funds of the Red Cross Societies, the necessity of whose work is beyond question. No other charity fête on this scale can be easily recalled which has quite the same significance, and we doubt if the instinct for pleasure has ever been more happily exploited for a high purpose than it was here.

### ST. MARYLEBONE GUARDIANS AND THE COAL SUPPLY.

The Guardians of the parish of St. Marylebone are endeavouring to enlist the support of other boards and local authorities in concerted action, so that a sufficient supply of coal at a reasonable price may be ensured for the coming winter. The circular-letter points out the enormous advance in price of all coal since the war began, and in view of the hardship imposed on public bodies and citizens the Government is requested to introduce legislation giving effect to the recommendation of the Departmental Coal Committee, advising the Government to take over the control and regulation of the coal supply, production, and distribution. Copies of the resolution passed by the Marylebone Guardians have been sent to the Prime Minister and local members of Parliament asking them to support the resolution in the House of Commons. The question of the supply of coal to our hospitals and infirmaries is one that demands attention, as even at the enhanced prices prevailing it is often difficult to get delivery. Whether a central store (as previously suggested) would meet the difficulty is doubtful, unless the Government also take action. It is asked whether, perhaps, the Committee of King Edward VII.'s Hospital Fund would support some action as suggested by the Marylebone authorities. If so, would the necessity of paying huge profits to some contractors probably be obviated? [Readers are also referred to p. 306.]

### THE HOSPITAL EGG.

Most institutional housekeepers will have realised that the new-laid egg has become an expensive luxury, albeit a necessity; and if those who should be able to speak are to be believed, the present prices will be doubled before Christmas. Hospitals therefore, where it is possible, may try to obtain eggs at a fairly reasonable price to "put down" a quantity for winter use. A correspondent who has had some experience with poultry has in past years made it a practice to preserve a number of eggs with great success (he says) in the following way. The eggs, as new laid as possible—not more than two days old, preferably only one—are placed in an earthenware vessel pointed end downwards, and a solution of "water glass" (obtainable from any drug stores and mixed according to instructions) poured over so as entirely to cover the eggs, care being taken, should any of the latter float, to remove them; a cover is placed on the vessel, which is allowed to stand in a cool place. Eggs thus "pickled" will remain perfectly fresh for six to nine months, or even longer; but if required for boiling the shells must be pierced, otherwise they will crack.

### A SUGGESTED ECONOMY IN THE EGG BILL.

The above suggestion of our correspondent that hospitals should take advantage of the present summer prices (such as they are) and pickle their eggs, lest worse prices should prevail during the winter, will strike different hospital officers in very different ways. For the fact is that the ways of hospitals in the matter of eggs are almost as varied as the ways of contractors' eggs with hospitals. Indeed, perhaps this is a libel on the eggs, although the practice of some hospitals in keeping small reserves of eggs seems to countenance the theory that fear of price or fear of flavour prevents their regular appearance in the diet of these institutions. For the small users of eggs the suggestion that they should lay in a supply in summer and pickle it according to our correspondent's recipe may or may not commend itself: the number of eggs they normally use is small. Or they may say they cannot get enough; if so, we suggest they should test our correspondent's optimism. Other large hospitals do not pickle, presumably because they can arrange a sufficient supply on what seems to them favourable terms. These we commend to reconsider their arrangements and contracts, as the present duty of economy demands. A more excellent way is suggested. Let it be tried, or, if not, at least criticised by our readers. One London hospital, we know, does pickle its eggs in summer if occasion requires. This is Charing Cross Hospital; and their views, therefore, on the above suggestion and recipe would be heard with special interest.

### NEEDLEWORK AND LINEN GUILDS AT CARDIFF.

The value of persistence during war-time, not only in the routine requirements of a hospital but also in all its auxiliary aids, was well illustrated at Cardiff last week by the annual meeting of the associates of the King Edward VII.'s Hospital Needlework and Linen Guilds. The meeting was exceptional in many respects, but it was gratifying to find that although the usual tea and garden party were dispensed with, on account of the war, there was no diminution in attendance. The meeting was presided over by Mrs. Henry Lewis, the retiring President of the Needlework Guild, who gracefully introduced the new President, Mrs. J. Herbert Cory. Lady Courtis, the President of the Linen Association, read a fairly satisfactory report, announcing that the value of the contributions received was £150, as compared with £158 last year, but that the money usually placed in the reserve fund had to be spent in the purchase of linen sheets. However, Sir William James Thomas had kindly promised to refund the £25 which she had spent in this way. Sir William's gift will have the effect of ensuring a record result on the year's working. Colonel Bruce Vaughan took the opportunity to enlist the sympathies of the Presidents, committee, ladies, and associates in the needs of the hospital, which had not yet been met. These specially appeal to the sympathies of womanhood, since they include a maternity ward, special wards for tuberculous patients, a new operating theatre, a convalescent home, and a hospital church.

### GERIATRICS.

The "first society ever organised" for the scientific study of senile conditions was inaugurated in New York on June 2, 1915, under the name of the New York Geriatric Society. The president is Dr. Robert Abraham, and the *New York Medical Journal* states the society's object to be the study of the causes of ageing and the diseases of advanced life, and the home and institutional care of the aged. There will be monthly meetings. The study of old age has already several text-books to its credit. In French we are reminded of Boy-Teissier, "Maladies des Vieillards"; Charcot, "Senile and Chronic Diseases"; and Ranzier, "Maladies des Vieillards"; while in English there are, for instance, the writings of Sir J. Crichton-Browne, "The Prevention of Senility"; J. Milner Fothergill, "Diseases of Sedentary and Advanced Life"; Professor Humphry, "Old Age"; Professor Saundby, "Old Age"; and Sir Hermann Weber, "Means for the Prolongation of Life." The study of senile decay has too often been undertaken by those who suffer from it; hence, perhaps, the claim of this new body to be the first in the field.

### THE PETER PAN OF FORTY.

Geriatrics is no doubt as proper an object of study as pediatrics, but, judging from recent popular literature on the subject, students of old age are apt to busy themselves with the modern equivalent of the philosopher's stone, that is a so-called scientific "health formula" as an elixir of life. For this they will inculcate a fixed diet, or forbid the use of starched collars, or say that it is wicked to wear wool; in short, run mad in their terror of death rather than devote themselves to scientific study of the subject. The result is that their works are unreadable except to people like themselves, and if the new Geriatric Society is not to add to this growing, and silly, literature, it must sternly set its face against the notion that we really desire death to be postponed more or less indefinitely. The myth of Tithonus is still too deep for many of us; and the reason, perhaps, why pediatrics has become an established study, while geriatrics is still hardly a study at all, is because no one has examined the diseases of childhood with the object of seeing if a formula could be found to avoid growing-up. Peter Pan may be a charming fellow, but he would not write instructive works on childhood because, being an eternal child, he is neither human nor a child at all, and therefore is placed at once outside the understanding of his subject. There are, however, Peter Pans of forty or thereabouts who do pretend to want to remain middle-aged for ever, and even write works to show how it can be done. Their works are as worthless as the desire which prompted them, so let the New York Geriatric Society be warned in time, and, having got over the fear of death, stick closely to the scientific side of the subject. For science does not reveal her most intimate secrets to the coward; any more than she does to mere callousness, or curiosity.

### A ROYAL ARMY DENTAL CORPS?

A suggestion was made not long ago that dentists should be required to attain a higher degree of medical knowledge than is at present required of them. The object was to ensure on their part a wider appreciation of the close relation that exists between oral hygiene and the maintenance of good general health. The importance of oral sepsis as an etiological factor in the pathology of many diseases is now an established maxim, but is not this a substantial reason for advocating an extended knowledge of dental surgery on the part of the medical practitioner rather than the converse suggestion above mentioned? When all is said and done, dentistry is a branch of surgery quite as truly as are the operations of adenoidectomy or circumcision. Over and over again it has happened that a practitioner, who has taken the trouble to remove septic stumps, and to treat the gingivitis present, has scored heavily in kudos and the patient in restored well-being. It is now being mooted in some quarters that the dental needs of the Army should be met by the formation of a Royal Army Dental Corps—a proposition which, if logically followed up, would lead to the formation of a Royal Army Orthopædic Corps and many others. Obviously assistance must be obtained for the making of dentures, etc., but there is no apparent reason why R.A.M.C. officers should not be required to attain some practical skill in dental surgery, as well as in other special branches of surgical work. These measures would surely suffice to provide for the ordinary needs of the Army.

### "PLACES OF SAFETY" UNDER THE MENTAL DEFICIENCY ACT.

The London County Council, who are responsible for the administration of the Mental Deficiency Act in the Metropolis, have failed so far to induce many of the Boards of Guardians to allow their institutions to be used as "places of safety" until the Council have found other institutions where the patients might be placed. In South London both Lambeth and Wandsworth declined to let the mentally afflicted be removed to their institutions; but in Southwark the concession was granted, to the cost of the Guardians, as eventually has been proved. A young girl at Balham came under the care of the officers of the London County Council responsible for the mentally afflicted, and it was deemed necessary, in the interests of the girl, that she should be placed in a "place of safety." As Lambeth and Wandsworth both refused to accept her, the only option was to take her to Newington, the institution of the Southwark Board of Guardians, as the nearest "place of safety to her home," though on the journey she passed the institutions of Lambeth and Wandsworth. The Southwark Board of Guardians felt naturally aggrieved at patients from other parishes in London being brought to their institution, and have made a strong protest to the London County Council. They naturally thought that when they gave the desired permission for their institutions to be used as "places of safety" the patients would come only from their own borough, little imagining they would be called upon to receive patients from the greater part of South London. The London County Council should take immediate steps to remedy this grievance, or they might find the Southwark Board of Guardians following the example of their neighbours at Lambeth and Wandsworth, and refusing to accept any patients at all.

### A SURCHARGE FOR ASSISTANCE AT OPERATIONS.

In our issue of December 26, 1914, we drew attention to the action of the auditor in surcharging two members of the Lexden and Winstree Guardians because they had sanctioned, without the consent of the Local Government Board, the payment to the workhouse medical officer of the expenses he had incurred in obtaining assistance for the performance of an operation on an inmate. We said the surcharge would no doubt be remitted on appeal, and this has proved to be the case. The Board holds that the reasons assigned by the auditor fail to support his decision. "Urgent and special cases may arise in which Guardians, on the advice of their medical officer, may consider that a serious operation of a kind that would usually be performed at a general hospital should be performed at the workhouse, which cannot be undertaken by the medical officer, or cannot be performed without the aid of another surgeon as well as an anæsthetist, and the Board are not prepared to hold that in a case of this type Guardians cannot engage assistance and incur reasonable expenditure without their sanction." It is satisfactory that this point has now been clearly settled. The decision does not, however, remove all the difficulties presented by these cases where immediate operations are required. They have to be done at once without as a rule any opportunity of consulting the Guardians, and the medical officer may incur liabilities which the Guardians may repudiate. This point did not arise in the present instance, but it is one upon which an indication of the views held by the Central Board would be valuable. The subject of operations in workhouses is one upon which the Board's regulations and instructional letters are now obsolete and urgently need revision.

### INFIRMARIES AND THEIR COAL SUPPLIES.

The Metropolitan Boards of Guardians are taking measures, during the summer months' decrease in the consumption of coal, to provide reserve stocks for the coming winter. In some instances special places have been taken to store coal, and, as a general rule, the contractors are falling in with the wishes of the authorities, and sending in good quantities to be ready when the shortage commences and the prices go up. In doing this they have only forestalled the action of the London County Council, who have circularised various public authorities suggesting they should adopt this course. The Departmental Committee of the London County Council have been requested to acquire and store within easy reach of London large stocks of household coal to be sold during the winter at prices, and under conditions, to be fixed in consultation with the Government to traders engaged in supplying small consumers. In order to carry this into effect it is necessary that those responsible for the administration of large institutions should take steps to see that their supplies are assured, or otherwise the London County Council may be called upon to supply to public authorities coal which would otherwise have been sold to the very poor at an exceedingly low price. In order to surmount the coal difficulties of the coming winter one way is for the public authorities to see that their supplies are fully replenished at the present time, so that later on the coal may be forthcoming for those really requiring it—that is to say, for those who have neither the means nor the room to acquire a large stock at the present time. For another suggestion see page 303.

### THE MONOTONY OF CONVALESCENCE.

We all realise this as a fact in the sick-room. We realise it less easily as a fact in the circumstances of a country mansion on the Downs, with numerous comrades and friends around us, to say nothing of a generous and sympathetic public outside. It is, therefore, well to be reminded how this monotony may press under the happiest institutional surroundings, and for this purpose the published appeal of Lord Killanin, as treasurer of the convalescent hospital, Woodcote Park, Epsom, is very forcible. Briefly he points out that his men "are up and about all day long," and that this normally happy process by itself is insufficient to secure unaided the complete restoration which they and we desire. He therefore, asks, in addition to many local offers of assistance in the way of providing lectures, entertainments, and concerts, for financial help to pay the incidental expenses even these offers necessitate, and also for someone to provide a concert or entertainment for the men in the afternoon or evening. The request is such a natural one that we are sure that the public will take pity on the sufferings incidental to unoccupied leisure, and send their gifts promptly either to the treasurer, or to Colonel Kilkelly, C.M.G., Commanding Officer at Epsom. Boredom is the last enemy to be overcome on the road to health, and, like most last enemies, it is one of the worst.

### NEGRO PATIENTS AT BRISTOL ROYAL INFIRMARY.

It is a trite saying that the Mother Country and the Dominions have been more united by the war than they would have been without it, but the illustration of this that comes home to hospital men is the sight of the wounded from the Dependencies of the Empire. In Lancashire, for instance, we have just received information of the number of wounded from remote parts of the world accommodated in one institution alone. That the hospitals must also be prepared to receive patients from the four corners of the world is seen by the recent arrival of eight negroes at Bristol Royal Infirmary. Under such circumstances surely this constitutes a record? The men were involved in the sinking of the Leyland steamship *Armenian*, which was torpedoed and sunk on June 28 by a German submarine. Mr. Page, the American Ambassador, has issued a statement concerning the loss of the ship, in which he states that Dr. Viso and three negroes are believed to have been picked up by the submarine. The men were twelve hours in lifeboats and on rafts before they were picked up by a Belgian trawler, which handed them over to two torpedo-boat destroyers, when they were brought into Bristol the next day, June 29. The *Armenian*, which was bound for Avonmouth, had 1,414 mules on board.

### INSANITY AND ECONOMICS.

The Report of the Inspector-General of the Insane, Western Australia, for the year ending December 31, 1913, is now to hand, and contains one or two matters of interest, which reflect the conditions of mental hospital work and its results in the State. There were 934 certified persons in this State who were principally accommodated in Claremont Mental Hospital, where no fewer than 920 out of the total were received. The increase for the year was 58 of both sexes, or eighteen more than in the preceding year. In other words, the ratio of the insane to the sane on December 31, 1913, in Western Australia was 2.92 per 1,000; the proportion is shown by tables to be slightly increasing, but the report states that it is considerably less here than in any other State with the exception of South Australia. It is interesting to note that the re-admissions only numbered 28, or 8.21 of the total admission, which the report records as "gratifying." Moreover the number of discharged was 90, of whom 76 were discharged recovered; the proportion of recoveries to admissions was 33.04 per cent. "Considering that the patients admitted to Claremont are not," says the report, "of the temporary type, this proportion of recoveries is very gratifying." The cost of maintenance is given as £54 2s. 9d. per head per year, a sum which includes "salaries, provisions, bedding, clothing, light, fuel"; but since the revenue, mainly from patients' fees and the dairy farm profits, has to be deducted from the gross cost, the actual net cost of maintenance per patient per annum is reduced to £44 6s. 5d. This throws an interesting light on the economic productivity of mental patients in Western Australia, a subject the general bearings of which aroused much interest in this country (in connection with the feeble-minded) at the time when the Mental Deficiency Act was passed. Our readers may recall that Dr. Rotherham and Miss Dendy, who subsequently were appointed members of the Board of Control, discussed in elaborate detail in the Special Number of The Hospital devoted to the practical administrative problems of the Mental Deficiency Act, how far patients can contribute to the cost of their support. With the attention now being paid, in spite of the war, to the treatment on a more or less comprehensive scale of all divisions of mental patients, the question of how far such patients can contribute towards the enormous cost of their segregation and supervision has become a pressing one, and any light to be gained from allied work in the Dominions is therefore to be welcomed.

### SANATORIUM ADVANTAGES FOR INFIRMARY CONSUMPTIVES.

Dr. Baly, medical superintendent of the Lambeth Infirmary, has presented to the Lambeth Board of Guardians a report of a visit he paid to the Grosvenor Sanatorium at Ashford, Kent. The institution, he points out, is well suited to the treatment of tuberculous patients, all of whom appeared to be contented and happy. Graduated work in the form of keeping the grounds in order was provided under the direction of the medical superintendent. This was a recognised form of treatment, and the fact that it was not provided at other sanatoria was a drawback he had previously reported to the board on several occasions. If it were possible to transfer all the chronic tuberculous patients in the infirmary to Ashford, the difficulties in connection with them, of which the board had full knowledge, would be solved. The patients would have to do a certain amount of work, and consent to be clean shaved, and it would be understood that any patient whose disease became worse in spite of treatment would be returned to the infirmary. He believed that the patients would be a great deal happier in an institution like that at Ashford than they were in the infirmary, and would be willing to remain there. Acting under the advice of Dr. Baly, the Guardians have decided to make arrangements for the removal of all the chronic tuberculous patients in their infirmary to Ashford as soon as vacancies can be found for them.

### POOR-LAW MEDICAL OFFICERS AND THE CAUSE OF REFORM.

The annual meeting of this Association was held in the Council Chamber of the British Medical Association on July 6, and was presided over by the President, Surgeon-General Evatt, C.B. The Council in their report pointed out that their work had been seriously hampered by the war. Some members were largely engaged in military duties, and the work of all had been much increased, so that the Council had met only twice. It had been impossible, in consequence, to push forward the cause of Poor-Law medical reform with the energy possible at other times. In the early part of the year an attempt was made by the Paddington Guardians to appoint a district medical officer, residing in his district, subject to a three months' notice to determine his office. The Council protested against this breach of the Consolidated Orders, with the result that the Local Government Board declined to sanction this arrangement indefinitely, but permitted it for a limited period, so that the Guardians might review the whole question of the provision of outdoor medical relief in the parish. The Council drew attention to the fact that in many Poor-Law institutions the medical officers have been expected to do much extra work, as a consequence of the displacement of inmates by sick and wounded soldiers, without any extra remuneration. The report goes on to say: "The matter is a very difficult one, as any protest against such a manifest injustice is likely to be regarded as unpatriotic, and it is necessary that all Poor-Law medical officers should rally round the Association and assist its executive to safeguard the rights of the service."

### NEW ORTHOPÆDIC HOSPITAL FOR WOUNDED SOLDIERS.

The Queen Mary's Convalescent Auxiliary Hospitals at Roehampton were opened last week for the benefit of wounded men, both officers and rankers, who, having lost limbs in the war, have been discharged from naval and military hospitals. The honorary secretary and treasurer is Mr. C. H. Kenderdine, who supplies forms of admission and other particulars on application at St. Stephen's House, Westminster. These hospitals, and it is understood that present funds allow of 150 beds being maintained for one year at Roehampton, are base convalescent hospitals for certain orthopædic cases and men suffering from the effects of amputation. Recognised as such by the naval and military authorities they should perform a useful work in assisting in- and out-patients to be properly fitted with mechanical and artificial limbs. Hospital men with experience of orthopædic "after-care" will need no reminding that one of the difficulties experienced in the work is the supply of the proper kind of limb, since each case requires individual and expert fitting. Expense and other difficulties stand in the way of ordinary hospital patients, for the after-care work connected with these institutions presents problems all its own. There is therefore need that the best advice and the most carefully devised limbs for each individual should be secured to every wounded man of the country's forces who stands in want of them. Queen Mary's Convalescent Auxiliary Hospitals should do a useful work in looking after such military patients and teaching them, as in practice is generally found necessary, how properly to use their artificial limbs. Such special orthopædic departments as are attached to the few, too few, ordinary hospitals which possess them, and the handful of orthopædic hospitals in the country appear to be insufficient to cope by themselves with this work. Is this another instance of the small part open to the special voluntary hospitals in catering for the wounded?

### HOSPITAL CHAPLAINS AND THE HOSPITAL CHURCH.

Shakespeare's notion (Was it not?) that a rose by any other name would smell as sweet is one of those paradoxes which is at once true and false. How true it is, there is no need to insist on. Where it is untrue, as less appreciated, is worth a moment's attention. Hospital men are provided with an instance of this in the proposal of a certain large hospital to establish a hospital church, which, we understand, has created more interest than would have been the case had the authorities decided prosaically to appeal for funds to open a chapel. In this connection, moreover, we may note that the amusement of convalescents is not the only thing of which those interested in their welfare feel them to be in need. The Archbishop of Canterbury, for instance, stated on Tuesday last that the men who had returned from the Front with a deepened sense of reality had "complained that there were not sufficient ministrations to help to develop their spiritual life." Is this the practical reason why hospitals with many wounded are hoping to establish the hospital church, and not the hospital chapel? In saying that the existing arrangements for spiritual ministrations to the men would have to be extended during the second year of the war, the Archbishop is not reported to have indicated the best way in which this could be done. We may, therefore, without presumption, suggest that there is hereby revealed a great opportunity for the hospital chaplains, in conjunction with hospital chairmen, to give the Archbishop the benefit of their unique experience in this labour of love. The hospital church, if it is to come, should come not only because existing accommodation in our mostly small hospital chapels is limited, but because also the chaplains are seeing how best the existing need can be met in such a way as not to leave on the hands of the hospital authorities a building which, after the war, is felt not to justify its scope and the energies now associating themselves with it. What are our chaplains' and chairmen's views?

### CHRISTMAS FESTIVITIES IN SUMMER.

Just as in the early days of recruiting "Tipperary" was the song of enthusiasm, so the magnificent effort of the hospitals, or rather of the public through the hospitals, to make the wounded as cheerful as their circumstances permit, is expressed by the cigarette and the gramophone. With the practical problems arising from the former we have dealt before, for, after all the change of practice involved in permitting cigarette-smoking in the wards was a typical part of the new administrative changes which hospital officers had to face. The presence of the gramophone in hospital is another fact that deserves to be recorded at this time for the benefit of the future hospital historian. Luckily, that cheerful instrument has been steadily improving its manners of late years, otherwise a cynic might almost have said that it made confusion worse confounded. The facts are, however, that it is there and liked. The men, taking the institutions as a whole in which they dwell, can never have enough of them; and the agony column, not to mention others ordinarily less interesting, is almost daily enlivened by appeals for their provision. We say "enlivened" because, while the gramophone, combined with the concerts and entertainments now becoming regular features of hospital life, is turning many afternoons into "festivals," those hospital officers who are responsible for organising Christmas festivities are learning from its popularity that men patients anyway find it more congenial to a sick-bed than peace and quiet. There are exceptions, of course. Happily, many soldiers in hospital are only slightly wounded, but the general experience seems to indicate that the mild criticisms heard now and again concerning Christmas festivities as a mistake are, on the whole, unfounded. They cheer the patients, and only a few would be without them. Let this, then, be remembered when Christmas again is celebrated in peacetime: decorations may perhaps be dispensed with, but not laughter-making noise.

### ITALY AND THE DRUG MARKET.

The steady advance in the prices of drugs continues, and each week the list of commodities affected seems to grow longer. The effect on the drug market of Italy's intervention in the war is now becoming more pronounced. Citric acid has advanced in price substantially, and in consequence citrates are much dearer. The cost of effervescent preparations, which are in such large demand at this season of the year, has also increased since citric acid and tartaric acid are largely used in their manufacture. The oils of lemon, bergamot, and orange are also advancing in price as a direct consequence of the Italian intervention. Corrosive sublimate, calomel, and other salts of mercury are again dearer, as a result of the further advance in the cost of quicksilver. The high price of opium is firmly maintained, and an advance in price seems most probable; the large demand for morphine and codeine continues, and any reduction in price seems to be out of the question. Antimony preparations are again dearer. A further advance in the price of cod-liver oil has taken place as a result of renewed purchasing by Germany. Mineralised methylated spirit and industrial spirit are substantially dearer. Chloroform and ether have both advanced in price. Further supplies of salicylates have arrived, and the position has been relieved somewhat, but no considerable reduction in price can be expected. The values of phenacetin and acetanilide are not so firmly maintained, but although reductions in the prices of these two products are not altogether unlikely, there appears to be no prospect of any substantial fall in value.

